# Physiological and Biochemical Mechanisms and Cytology of Cold Tolerance in *Brassica napus*

**DOI:** 10.3389/fpls.2020.01241

**Published:** 2020-08-12

**Authors:** Weiliang Qi, Fei Wang, Li Ma, Ze Qi, Songqing Liu, Cun Chen, Junyan Wu, Ping Wang, Cairong Yang, Yong Wu, Wancang Sun

**Affiliations:** ^1^ College of Agronomy, Gansu Agricultural University, Lanzhou, China; ^2^ Gansu Provincial Key Laboratory of Arid land Crop Science, Gansu Agricultural University, Lanzhou, China; ^3^ Key Laboratory of Crop Genetics Improvement and Germplasm Enhancement of Gansu Province, Lanzhou, China; ^4^ College of Chemistry and Life Science, Chengdu Normal University, Chengdu, China; ^5^ College of Metallurgy, Northeastern University, Shenyang, China

**Keywords:** *Brassica napus*, cold stress, super-oxide anion (O_2_^−^), physiological index, biochemical index, cytology

## Abstract

Cold damage has negatively impacted the yield, growth and quality of the edible cooking oil in Northern China and *Brassica napus* L.(rapeseed) planting areas decreased because of cold damage. In the present study we analyzed two *Brassica napus* cultivars of 16NTS309 (highly resistant to cold damage) and Tianyou2238 (cold sensitive) from Gansu Province, China using physiological, biochemical and cytological methods to investigate the plant’s response to cold stress. The results showed that cold stress caused seedling dehydration, and the contents of malondialdehyde (MDA), relative electrolyte leakage and O_2_^−^ and H_2_O_2_ were increased in Tianyou2238 than 16NTS309 under cold stress at 4°C for 48 h, as well as the proline, soluble protein and soluble sugars markedly accumulated, and antioxidant enzymes of peroxidase (POD), superoxide dismutase (SOD), and catalase (CAT) were higher in 16NTS309 compared with in Tianyou2238, which play key roles in prevention of cell damage. After exposure to cold stress, the accumulation of the blue formazan precipitate and reddish brown precipitate indicated that O_2_^−^ and H_2_O_2_, respectively, were produced in the root, stem, and leaf were higher than under non-cold conditions. Contents of O_2_^−^ and H_2_O_2_ in cultivar Tianyou2238 were higher than 16NTS309, this is consistent with the phenotypic result. To understand the specific distribution of O_2_^−^ in the sub-cellular, we found that in both cultivars O_2_^−^ signals were distributed mainly in cambium tissue, meristematic cells, mesophyll cytoplasm, and surrounding the cell walls of root, stem, leaves, and leaf vein by morphoanatomical analysis, but the quantities varied. Cold stress also triggered obvious ultrastructural alterations in leaf mesophyll of Tianyou2238 including the damage of membrane system, destruction of chloroplast and swelling of mitochondria. This study are useful to provide new insights about the physiological and biochemical mechanisms and cytology associated with the response of *B. napus* to cold stress for use in breeding cold-resistant varieties.

## Introduction

Rapeseed (*Brassica napus* L.) is an important oilseed crop in the world with sown area exceeding 7 million hectares in China. Rapeseed is divided into winter and spring types, most of which is winter type with more than 90% (Yan et al., 2019). Winter *B. napus* is mainly distributed in the Huang - Huai River and Yangteze River basin, but the same *B. napus* varieties were difficult to survive in northern China (Pu et al., 2019b). As a winter cover crop, *B. napus* not only eliminates the dust source of the damaging sand storms in northern China, but are also economically beneficial in the dry and cold regions of northwest China. In China, cold affected area of winter rape of the *B. napus* accounts for 77.8% and planting areas has been reduced by about 10.9% because of cold damage, such as an extremely cold weather, ice rain and heavy snow. Cold damage has negatively impacted the yield, growth, and quality of the edible cooking oil in China (Sun et al., 2007; Zhang et al., 2008; Sun et al., 2010; Huang et al., 2019). Many studies also shown that *B. rapa* has better cold resistance than *B. napus* (Sun et al., 2007; Sun, 2013), and several *B. rapa* species have been successfully cultivated in northern China. Thus, it is urgent to develop more cold resistant varieties of *B. napus*. In order to survive in harsh natural environment conditions, plants have evolved complex mechanisms, such as accumulation of compatible osmolytes, stimulation of efficient enzymatic and non-enzymatic antioxidative system (Dietz et al., 2006; Meyer et al., 2012; Noctor et al., 2014). Cold stress lead to the accumulation of excess reactive oxygen species (ROS) such as super-oxide anion (O_2_^−^), hydrogen peroxide (H_2_O_2_), oxygen singlet oxygen (^1^O_2_) and the hydroxyl radical (HO·)(Asada and Takahashl, 1987; Mittler et al., 2004; Tripathi et al., 2017; Banerjee et al., 2018; Huang et al., 2019) which cause oxidative stress and lead to a variety of biochemical and structural changes and cell death (Apel and Hirt, 2004; Vacca et al., 2004; Vacca et al., 2006; Lushchak, 2014; Marcec et al., 2019). ROS signaling has been studied in plant cells, but still unclear. Gill and Tuteja (2010) reported that chloroplasts of photosystem I and II (PSI and PSII), and mitochondria of complex I, ubiquinone and complex III of electron transport chain (ETC) are the major sites for the generation of O_2_^−^ (Zhao et al., 2018). But also are ‘deliberately’ produced at plasma membrane level, or extracellularly in plants such as class cell wall-peroxidases, germin-like oxalate oxidase,s and amine oxidases and plant NADPH oxidases, which are by far the most studied O_2_^−^ producing enzymes (Apel and Hirt, 2004; Miller et al., 2010; Nanda et al., 2010; Gilroy et al., 2016; Singh et al., 2017). As diffusible and short-lived molecules, O_2_^−^ were unable to transport across the membrane’s lipid bilayer, but O_2_^−^ could be converted into hydrogen peroxide (H_2_O_2_) by variety of reactions (Gilroy et al., 2016; Toyota et al., 2018; Singh et al., 2019) that readily transferred across membranes passively. We can therefore envision ROS (O_2_^−^, H_2_O_2_, etc.) signaling as a dynamic process that occurs within cells between different organelles, as well as between cells over long distances (Mittler et al., 2011). More importantly, ROS (O_2_^−^, H_2_O_2_, etc.) can regulate a broad range of biological processes (Mittler et al., 2004; Mittler et al., 2011). Suzuki et al. (2011) reported that 345 or 97 transcripts are up-regulated specifically by H_2_O_2_ or O_2_^−^, respectively, indicating that distinct signaling pathways were activated by these different ROS (O_2_^−^, H_2_O_2_, etc.). Thus, localizing the O_2_^−^ signal at the sub-cellular is essential for us to gain deep knowledge of the mechanisms of O_2_^−^ production, transduction of O_2_^−^ derived signals, and especially the communication and interaction between different subcellular compartments in O_2_^−^signaling.

Our research group studied the feasibility of expanding winter rapeseed northwards into cold regions in northwestern China and bred new *B. napus* 16NTS309 having a strong cold tolerance, which could over winter in the 36°73′N area at an altitude of 1,517 m. These are the essential *B. napus* germplasm resources having strong cold tolerance levels used for breeding in northern China. Although the physiology, proteomics, and micRNA of cold tolerance of winter *B. napus* and *B.rape* have been studied (Ma et al., 2019; Pu et al., 2019b), but the cellular mechanisms underlying cold tolerance and resistance (Møller and Sweetlove, 2010) remain largely unknown. In this study, we address several key questions. Where do O_2_^−^ come from and are there differences in the accumulation of O_2_^−^ in *B. napus* of root, stem, leaves, and leaf vein? Do O_2_^−^ diffuse in the cytoplasm of cells and, if so, to what degree? What Kinds of modifications in plant growth, ultrastructural modifications, and anti oxidative response to cold stress in *B. napus*? The aim is to provide new insights about the physiological and biochemical and cytology mechanisms associated with the response of *B. napus* to cold stress for use in breeding cold-resistant varieties.

## Materials and Methods

### Plant Material

The *B. napus* cultivar 16NTS309 (strongly resistant to cold damage) and Tianyou 2238 (cold sensitive) provided by the Key Laboratory of Crop Genetics Improvement and Germplasm Enhancement of Gansu Province, Lanzhou. *B. napus* of 16NTS309 and Tianyou2238 were grown on experimental farm of Gansu Agricultural University of Lanzhou (36°73′N, altitude 1,517 m). The experiment was set up as a split-plot design with 3 replicates. The area of single plot was 4m^2^, furrowing seeding (spacing planting distance 8–10 cm). Sowing seeds in middle of September. The number of seedlings before overwintering stage and after overwintering datas was counted. The overwintering rates (%) = number of seedlings after overwintering stage/total number of seedling plants by 100%.

In the laboratory, seeds were also germinated in 12 × 8 hole float tray (60 × 40 × 8 cm) and pots were filled with a 3:1 mixture of nutritional soil and vermiculite, and are grown in an illumination incubator with normal conditions (25^◦^C, day/night temperature, 16 h photoperiod, intensity of illumination 6000 Lx). Until the plants grew to the four-leaf stage, then 16NTS309 and Tianyou2238 seedlings were divided into two groups. One group was transferred to normal conditions (25^◦^C, day/night temperature, 16 h photoperiod, intensity of illumination 6000 Lx) and kept for 48 h as a non-cold control. The other group was placed into a pre-cooling incubator for cold treatment at 4°C for 48 h (4^◦^C, day/night temperature, 16 h photoperiod, intensity of illumination 6000 Lx). The experiment was repeated three times.

### Plant Callus System

A plant callus system of *B. napus* cultivar Tianyou2238 (cold sensitive) was established using the leaves. To characterize callus growth, seeds were surface sterilized with 0.5% hypochlorous acid (HClO) for 8 min and germinated in the dark for 48 h, then grown in 200 ml flasks containing 50 ml of liquid murashige and skoog (MS) medium. The resultant embryos were placed on callus-generating medium containing MS salts supplemented with 1mg.L^−1^ 2, 4-dichlorophenoxyacetic acid (2,4 - D) and 1 mg.L^−1^ 6-benzylaminopurine (6 - BA) and adjusted to pH 6.0, prior to autoclaving. The embryo cultures were maintained at 25°C in low light conditions. Then 100 calluses were obtained after 8 days of incubation. The calluses of cultivar Tianyou2238 were divided into two groups: one was maintained at 25°C as the non-cold control and the other was subjected to cold stress at 4°C (Li et al., 1998).

### Physiological Index Method

After the cold treatment, we used fresh materials immediately measured the relative electrolyte leakage by a digital conductometer DDS11A (Leici Instrument Factory, Shanghai, China) according to Bajji et al. (2002). O_2_^−^ was measured as described by Elstner and Heupel (1976). H_2_O_2_ was measured as described by Sergiev et al. (1997). The content of soluble sugar, soluble protein, malondialdehyde (MDA), proline (Pro), superoxide dismutase (SOD), peroxidase (POD), and catalysis (CAT) measured using the frozen sample. Soluble sugar content was measured as described by Buysse and Merckx (1993). Soluble protein content was measured using coomassie brilliant blue staining (Zou, 2000). MDA was determined by the thiobarbituric acid (TBA) reaction (Dhindsa et al., 1981) and the content of Pro was measured by the sulfosalicylic acid-acid ninhydrin method (Bates et al., 1973). The activity assay of SOD, POD, and CAT were recorded according to Wu et al. (2003). All physiological results were repeated for three times and the data were analyzed by one-way analysis of variance (ANOVA) using SPSS 20.0 (IBM Corp., Armonk, NY, USA) to detect significant differences between both treatments (P < 0.05). Results were plotted with Adobe Photoshop CC 2018 (Adobe Inc., San Jose, CA, USA).

### Detection of H_2_O_2_ and O_2_^−^

Seedlings were placed in the test tubes and immersed in 3, 3ʹ-Diaminobenzidine (DAB) and Nitrotetrazolium blue chloride (NBT) staining solution to detect H_2_O_2_ and O_2_^−^ as described by Kumar et al. (2014) and Wu et al. (2015). Plants were immersed for 8 h with DAB and NBT staining solution that solution should be placed away from light. After infiltration, the stained plants were bleached in an acetic acid:glycerol: ethanol (1:1:3, v/v/v) solution at 100°C for 10–20 min, then stored in 95% (v/v) ethanol until scanned. The experiment was repeated six times.

### Transmission Electron Microscopy

For ultrastructural analysis of leaf tissue, samples were fixed with 2.5% gluteraldehyde fixative in 0.1 M phosphate buffered saline (PBS) at pH 7.4 for 2 h under vacuum. Then, the samples were rinsed repeatedly with PBS before and after secondary fixing with 2% osmium tetroxide. The fixed samples were polymerized in epoxy resin, in an oven at 70°C for a day. The resulting polymerised resin blocks were sectioned at 60–100 nm thickness with an ultramicrotome and mounted on copper grids for observation with a transmission electron microscope (STEM CM12, Philips). Digital micrographs were captured and stored (Stefanowska et al., 2002; Ali et al., 2018a).

### Tissue Section

NBT reacts specifically with superoxide and forms a blue formazan precipitate. The deepest root, stem, and leaf samples from cultivars 16NTS309 and Tianyou2238 that had blue formazan precipitate areas, were sectioned and used for the morphoanatomical analysis. The samples were fixed in formalin acetoalcohol (FAA) 50%, softened using 20% ethylenediamine, and embedded in paraffin. The samples were sectioned on a rotary microtome and stained with PAS-naphthol yellow or saffron-solid green. Slides were mounted with synthetic resin and images were captured using a digital image acquisition system.

### Chromosome Preparation

Seedlings of cultivars 16NTS309 and Tianyou2238 from winter oilseed rape (*B. napus*) were germinated on moist filter paper until the primary roots were 2–3 cm long. Then root tips were removed in 2% (w/v) cellulase (Calbiochem) and 20% (v/v) pectinase (Sigma Aldrich Corp., St Louis, MO, USA) in enzyme mixture for 40 min at 37°C. Chromosomes were prepared using a method adapted from Snowdon et al. (2002).The chromosomes were finally counter stained with DAPI (4, 6 - diamidino - 2 - phenylindole) solution (Vector Laboratories, Inc., Burlingame, CA, USA). The images were captured with an Olympus BX-51 (Olympus Corp., Tokyo, Japan) fluorescence microscope coupled to a Photometric SenSys Olympus DP70 CCD camera (Qi et al., 2016).

## Results

### Karyotype Analysis

**Figure 1:**
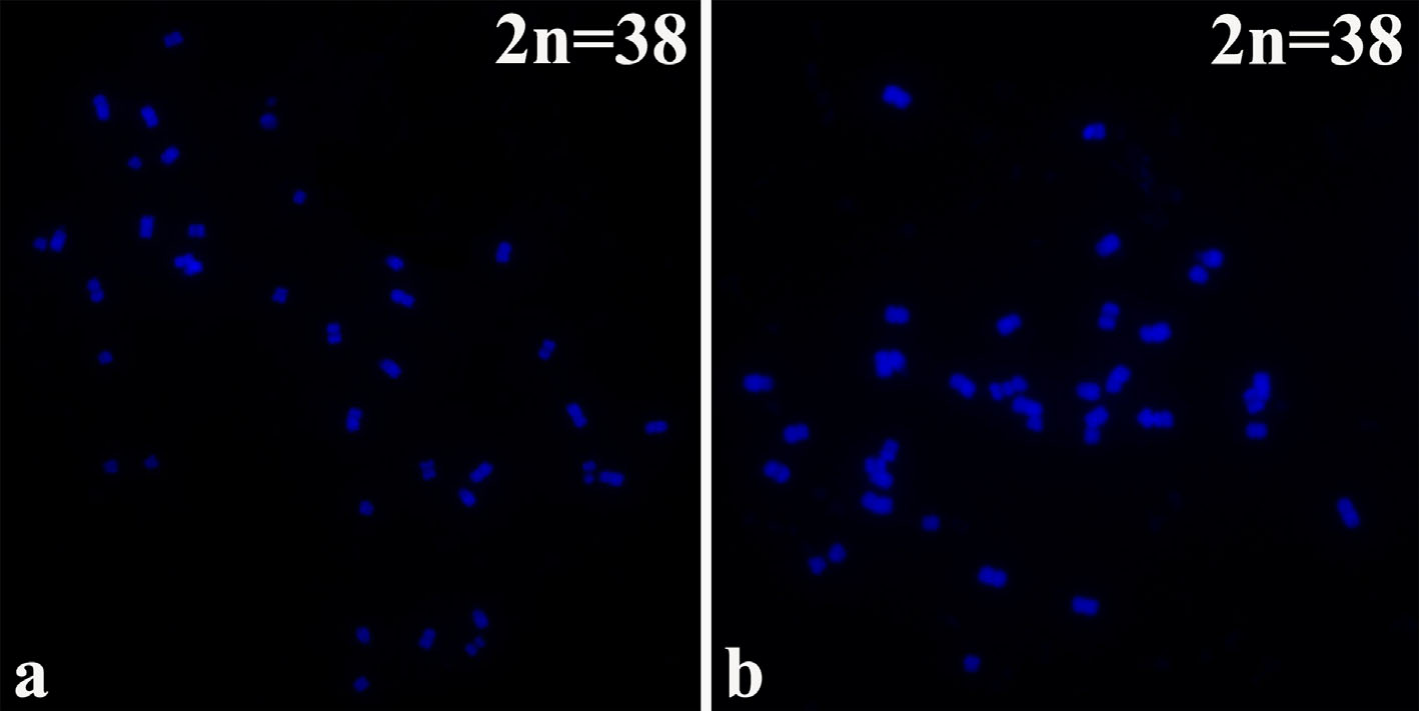
The chromosomes were counter stained with the DAPI solution. *B. napus* cultivars of **(A)** 16NTS309 and **(B)** Tianyou2238.

*B. napus* (2n=38; AA + CC) originated from the spontaneous hybridization of *B. rapa* (2n=20; AA) and *Brassica oleracea* L. (2n=18; CC) and contains the entire diploid chromosome sets of both parental genomes. The chromosomes of the highly homoeologous A genome of *B. rapa* and C genome of *B. oleracea* diverged relatively recently from a common ancestor (Lagercrantz and Lydiate, 1996). The 16NTS309 and Tianyou2238 cultivars, which belong to the tetraploid *B. napus* line (2n=4X=38; AACC) as determined by chromosome karyotype analysis (**Figures 1A, B**), were originally produced in our laboratory (Research Institute, Gansu Agricultural University, Gansu, China).

### Morphological and Physiological Responses to Cold Stress

**Figure 2:**
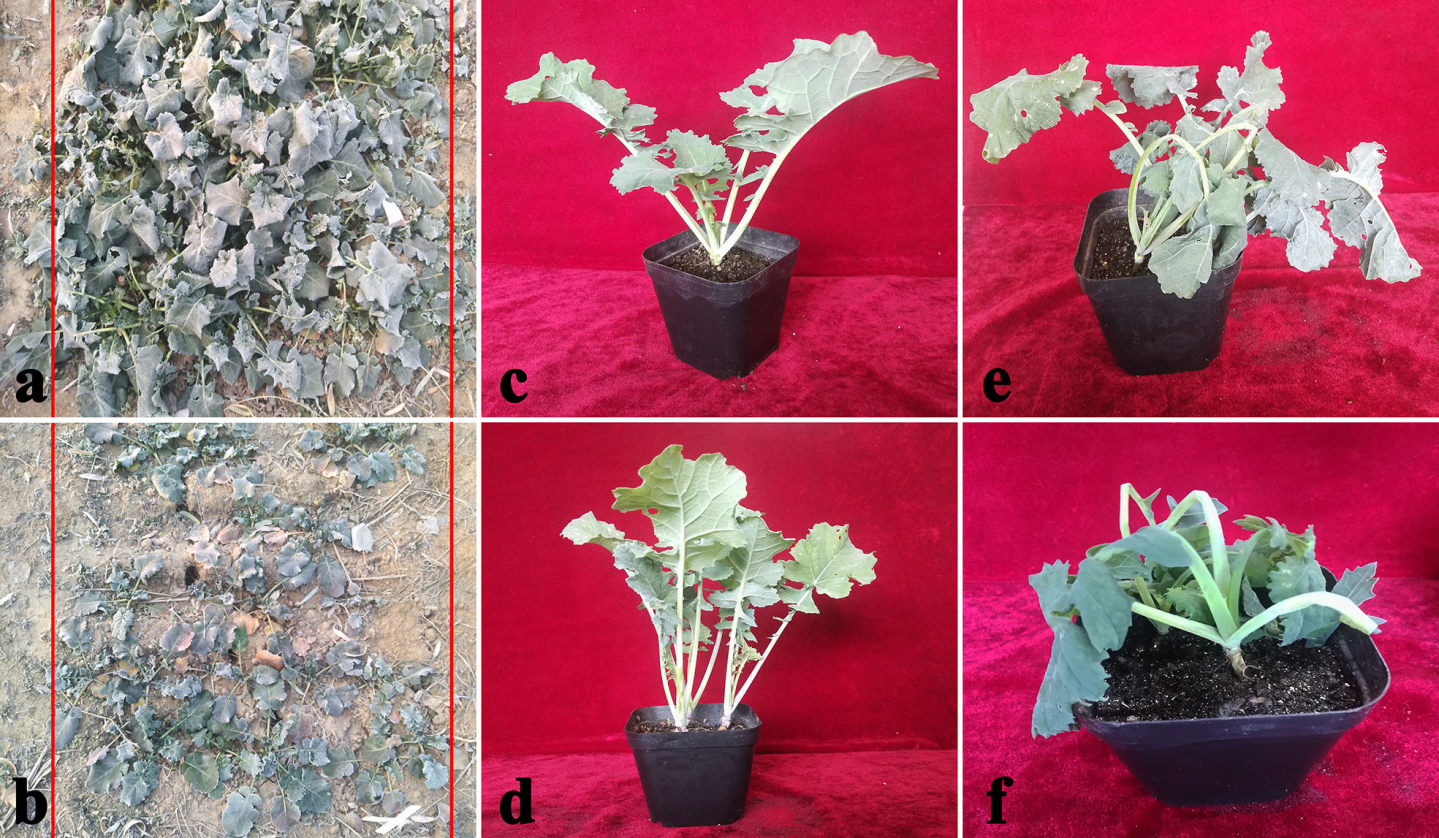
Phenotypic symptoms of cultivars 16NTS309 **(A, C, E)** and Tianyou2238 **(B, D, F)** in response to cold stress. Morphological characteristics of *B. napus* in December in the natural environment **(A, B)**, representative phenotypes of the plants under non-cold **(C, D)**, and cold stress conditions **(E, F)**.

**Figure 3:**
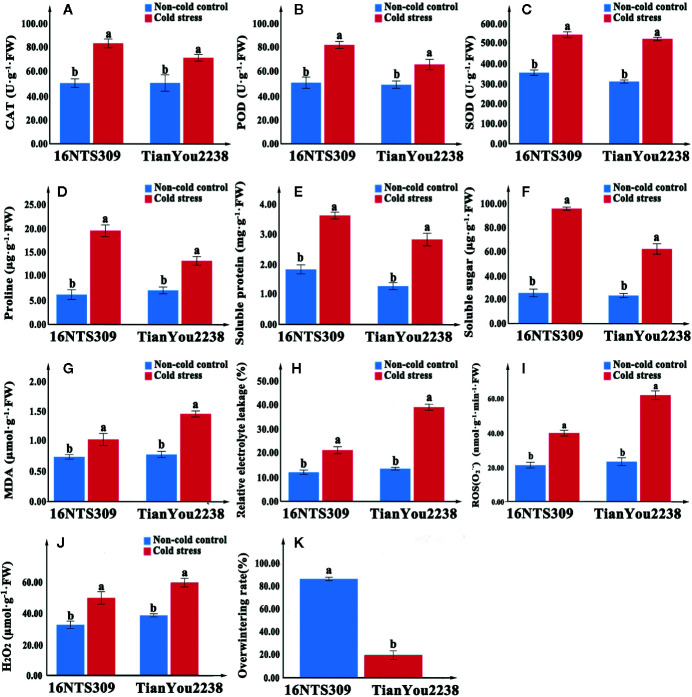
The activity of catalase (CAT) **(A)**, peroxidase (POD) **(B)**, superoxide dismutase (SOD) **(C)**, proline **(D)**, protein **(E)**, soluble sugars **(F)**, malondialdehyde (MDA) **(G)**, relative electrolyte leakage **(H)**, O_2_^−^ **(I)**, and H_2_O_2_ **(J)** content accumulation in leaves of 16NTS309 and Tianyou2238 under cold stress at 4°C for 48h. Overwintering rates was recorded in natural environment. Average overwintering rstes **(K)** in plants of 16NTS309 and Tianyou2238 under harsh winter conditions. The majuscules indicate a significant difference (P<0.05) for the data of the cold stress treated samples compared with non-cold stressed samples. The different small letters indicate significant differences at p < 0.05. The mean values were calculated from three biological replicates. Error bars denote standard error of the mean.

Two *B. napus* varieties were used as the materials. In order to compare the difference of cold tolerance between these materials in the natural environment, we observed the morphology of seedlings before overwintering (**Figures 2A, B**) in test fields of Lanzhou (36°73′N, altitude 1,517m) and recorded statistical overwintering rate. The strong winter *B. napus* of 16NTS309 could survive over winter in Lanzhou with average overwintering rates of 85.38%, which was higher than Tianyou2238 (**Figure 3K**).

Under normal conditions, two B. napus varieties were grown well (**Figure 2C, D**). After 48 h of cold stress at 4°C, 16NTS309 injury was minimal (**Figure 2E**), while the leaves of Tianyou2238 were showing marked wilting and chlorosis due to injury (**Figure 2F**). Under cold stress, the relative electrolyte leakage, MDA, O_2_^-^, H_2_O_2_, soluble protein, proline, and soluble sugars content of both cultivars increased compared with their content under non-cold conditions. However, relative electrolyte leakage (**Figure 3H**), MDA (**Figure 3G**), O_2_^-^ (**Figure 3I**), H_2_O_2_ (**Figure 3J**), increased by 187.33%, 84.81%, 170.49% and 53.84%, respectively, in Tianyou2238 which were higher than in 16NTS309. The soluble protein, proline, and soluble sugars in 16NTS309 were increased by 94.62%, 219.14%, and 271.3%, respectively, which is higher than they were in Tianyou2238. The activity of SOD, POD, and CAT also was higher in 16NTS309 compared with in Tianyou2238. These indexes were increased by 52.99% (**Figure 3C**), 60.39% (**Figure 3B**), and 65.92% (**Figure 3A**), respectively.

### Morphological and Anatomical Evaluation of O_2_^−^ and H_2_O_2_

**Figure 4:**
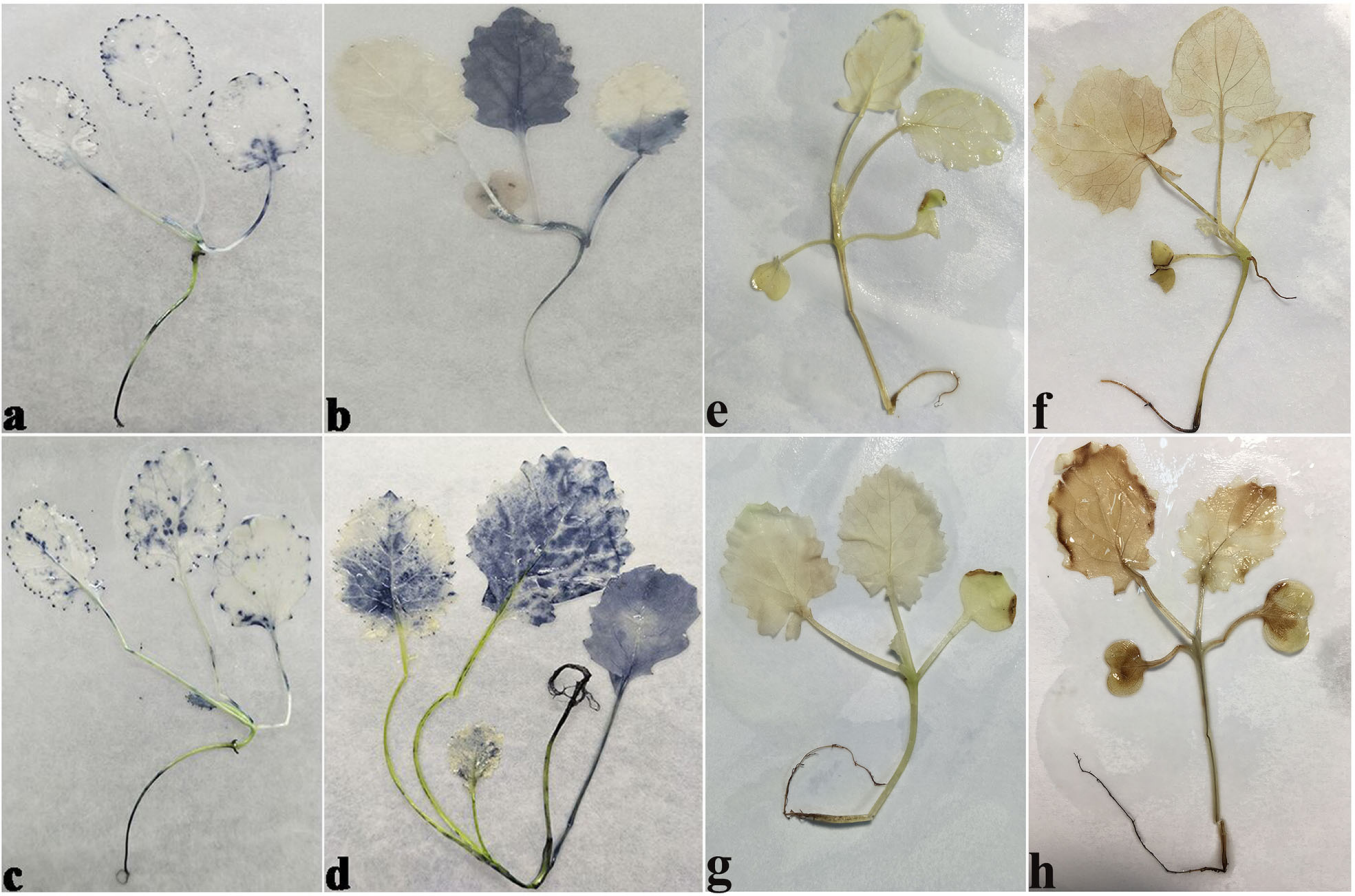
The O_2_^−^ **(A-D)**, H_2_O_2_ **(E-H)** levels in seedlings stained with Nitrotetrazolium blue chloride (NBT) and 3, 3ʹ-Diaminobenzidine (DAB) of cultivars 16NTS309 and Tianyou2238 under non-cold **(A, C, E, G)** and cold stress **(B, D, F, H)** conditions. *B. napus* cultivars 16NTS309 **(A, B, E, F)** and Tianyou2238 **(C, D, G, H)**.

NBT specifically reacts with O_2_^−^ and forms a blue formazan precipitate. DAB is oxidized by H_2_O_2_ in the presence of peroxidases and produces reddish brown precipitate. The accumulation of the blue formazan precipitate and reddish brown precipitate indicated the production of the O_2_^−^ and H_2_O_2_ in the root, stem, and leaf. Under normal conditions, seedlings of cold-tolerant cultivar 16NTS309 (**Figures 4A, E**) and cold sensitive cultivar Tianyou2238 (**Figures 4C, G**) also produce O_2_^−^ and H_2_O_2_ concentrations were in root, stem, and leaf. After exposure to cold stress, Tianyou2238 (**Figures 4D, H**) accumulated more O_2_^−^ and H_2_O_2_ than 16NTS309 (**Figures 4B, F**).

**Figure 5:**
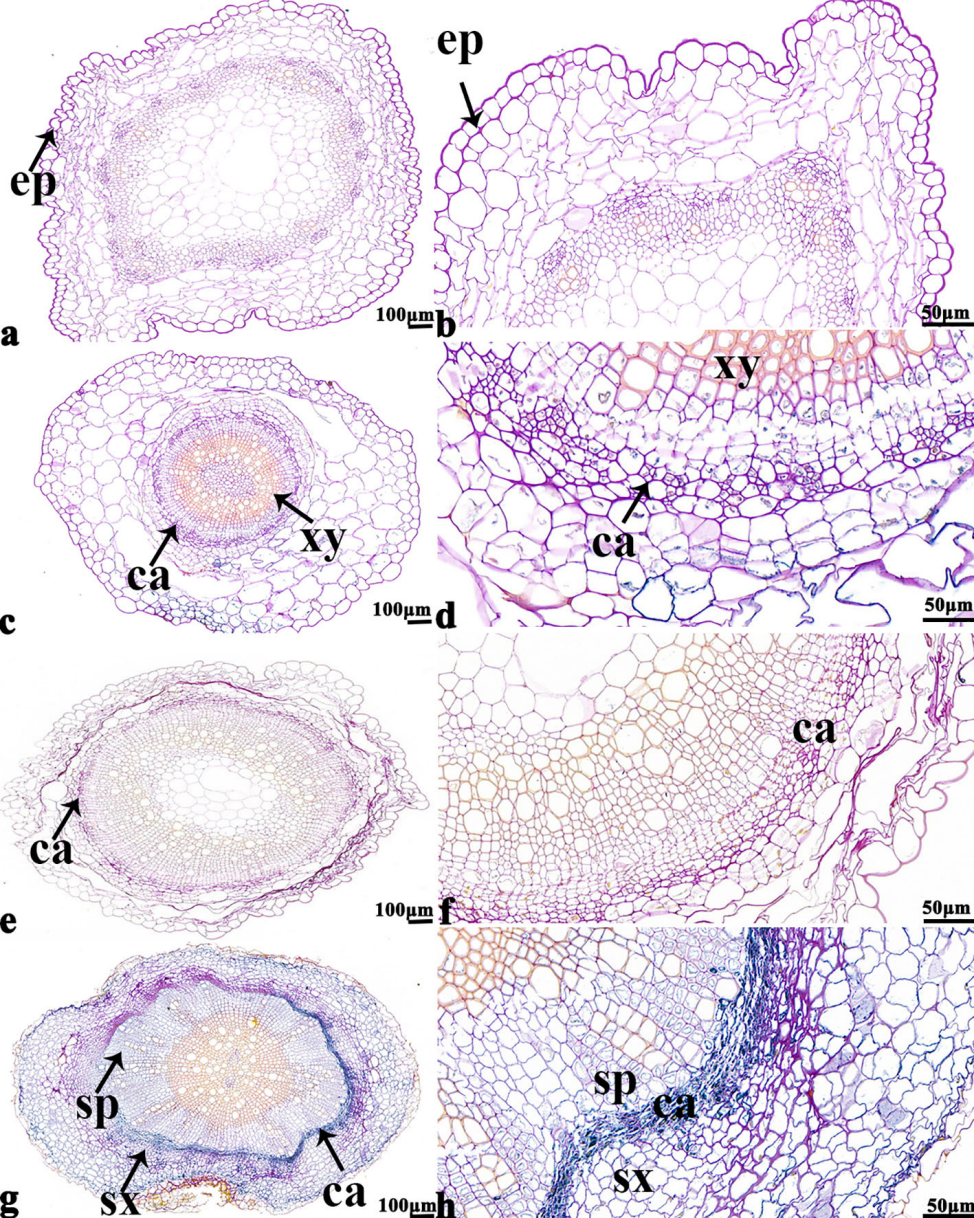
*B. napus* cultivars 16NTS309 and Tianyou2238 under non-cold **(A, B, E, F)** and cold stress **(C, D, G, H)** conditions for 48 h. The deepest root samples with blue formazan precipitate areas in 16NTS309 **(A–D)** and Tianyou2238 **(E–H)** were sectioned for morphoanatomical analysis. ep, epidermis; ca, cambium; sp, secondary phloem; sx, secondary xylem. Scale bars = 50 µm and 100 µm.

**Figure 6:**
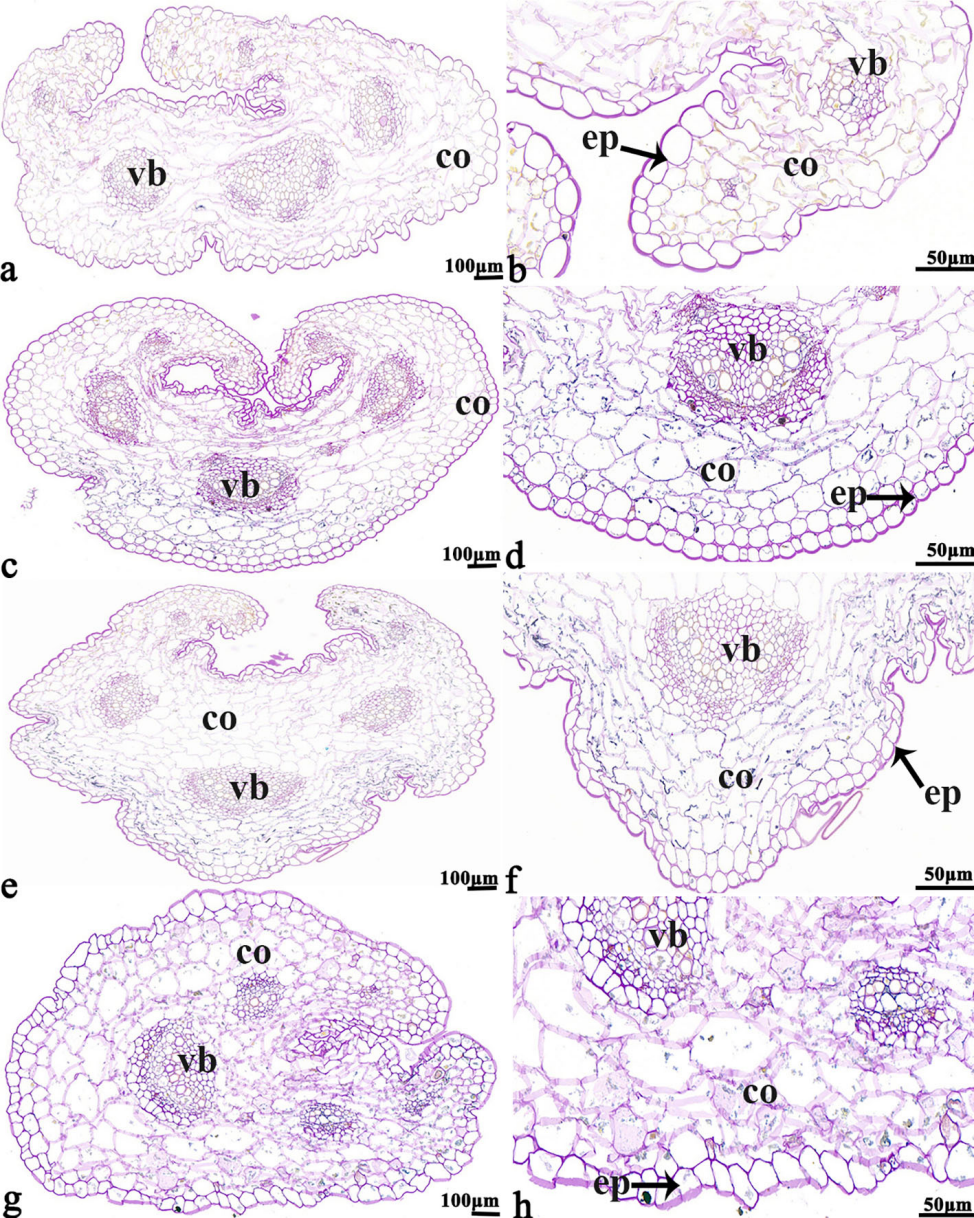
*B. napus* cultivars 16NTS309 and Tianyou2238 under non-cold **(A, B, E, F)** and cold stress **(C, D, G, H)** conditions for 48 h. The deepest stem samples with blue formazan precipitate areas in 16NTS309 **(A–D)** and Tianyou2238 **(E–H)** were sectioned for morphoanatomical analysis. vb, vascular bundle; co, cortex; ep, epidermis. Scale bars = 50 µm and 100 µm.

**Figure 7:**
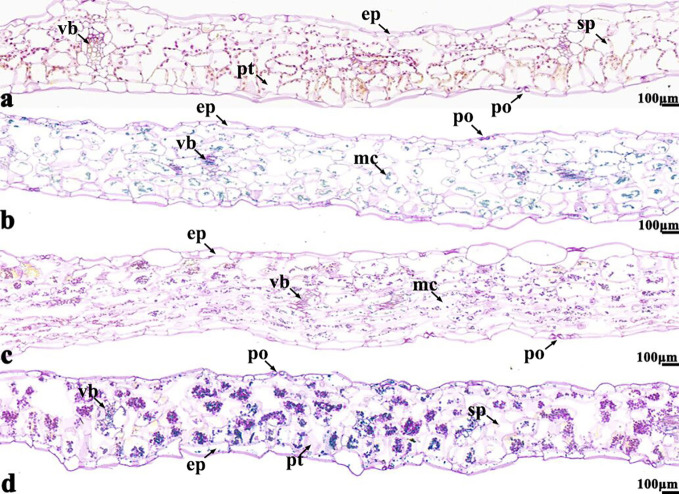
*B. napus* cultivars 16NTS309 and Tianyou2238 under non-cold **(A, C)** and cold stress **(B, D)** conditions for 48 h. The deepest leaf samples with blue formazan precipitate areas in 16NTS309 **(A, B)** and Tianyou2238 **(C, D)** were sectioned for morphoanatomical analysis. po, pore; ep, epidermis; sp, spongy parenchyma; pt, palisade tissue; vb, vascular bundle; ep, epidermis. Scale bars = 100 µm.

**Figure 8:**
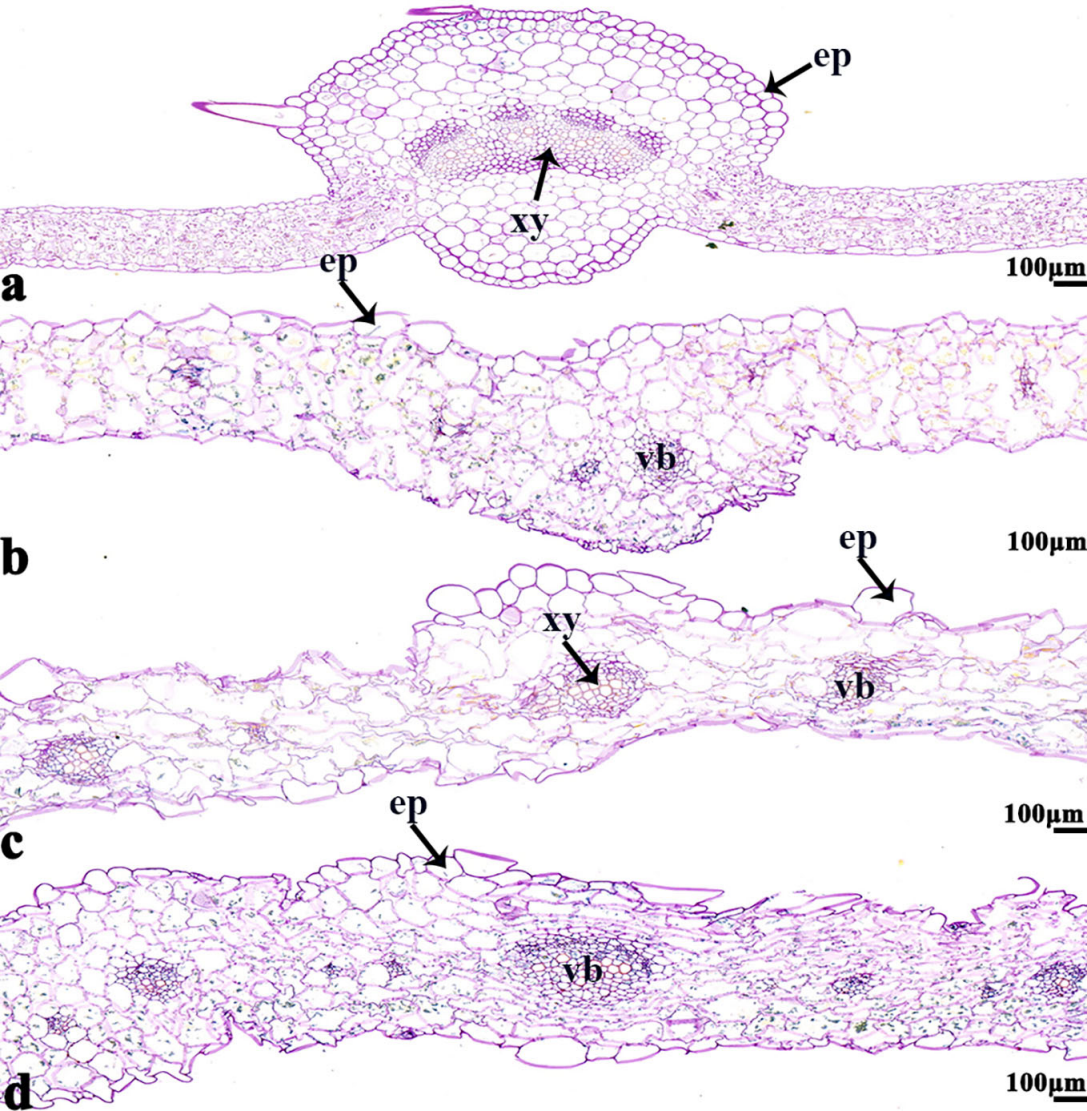
*B. napus* cultivars 16NTS309 and Tianyou2238 under non-cold **(A, C)** and cold stress **(B, D)** conditions for 48 h. The deepest leaf vein samples with blue formazan precipitate areas in 16NTS309 **(A, B)** and Tianyou2238 **(C, D)** were sectioned for morphoanatomical analysis. ep, epidermis; xy, xylem; vb, vascular bundle. Scale bars = 100 µm.

The NBT stained blue sections of root, stem, leaf vein, and leaf samples from 16NTS309 and Tianyou2238 used for the morphoanatomical analysis were selected for histological observation. The results showed that in the roots, the epidermis, secondary phloem, secondary xylem, cambium, and vascular cylinder had wide parenchymatous rays (**Figure 5**). The primary structures of the stems, the epidermis and cortex, developed notable vascular cylinders (**Figure 6**). In the leaves, the upper epidermis, lower epidermis, vein, stomatal palisade tissue, and spongy tissue showed starch accumulation (**Figure 7**). The veins contain epidermis and vascular bundles (**Figure 8**). Significant differences in O_2_^−^ production were found between the two cultivars and between the treatments. However the cambium, which is located between the phloem and xylem in roots, accumulated a lot of blue polymerization product after cold stress in both cultivars. Leaves mesophilic cells near the upper epidermis are cylindrical, forming the palisade tissue and mesophyll cells near the lower epidermis are irregular in shape and loose in arrangement, which were contains more chloroplasts. A greater O_2_^−^ content accumulated in palisade tissue and mesophyll cells compared with that in other leaf cells. This result indicated that chloroplasts play an active role in the production of O_2_^−^ (**Figures 7B, D**). In other mesophyll tissue cells, O_2_^−^ signals also were detected in mesophyll cytoplasm and surrounding cell walls, but the quantity in the two cultivars varied.

### Ultrastructural Analysis

**Figure 9:**
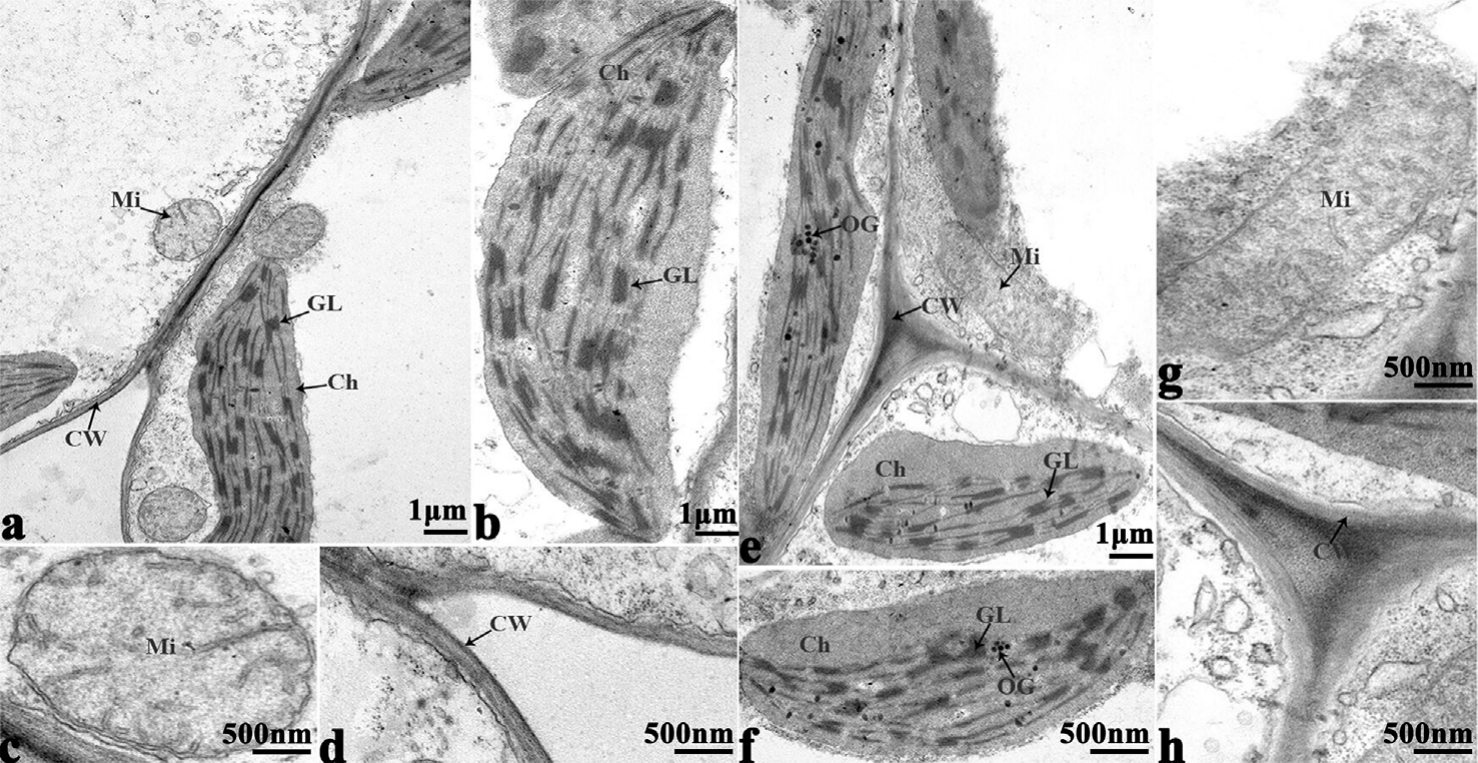
Transmission electron micrographs of leaf ultrastructure from cultivar 16NTS309 under non-cold **(A–D)** and cold stress **(E–H)** conditions for 48 h. Mi, mitochondria; Ch, chloroplast; GL, granum lamellae; OG, osmiophilic globule; CW, cytoderm.Scale bars = 1 µm and 500 nm.

**Figure 10:**
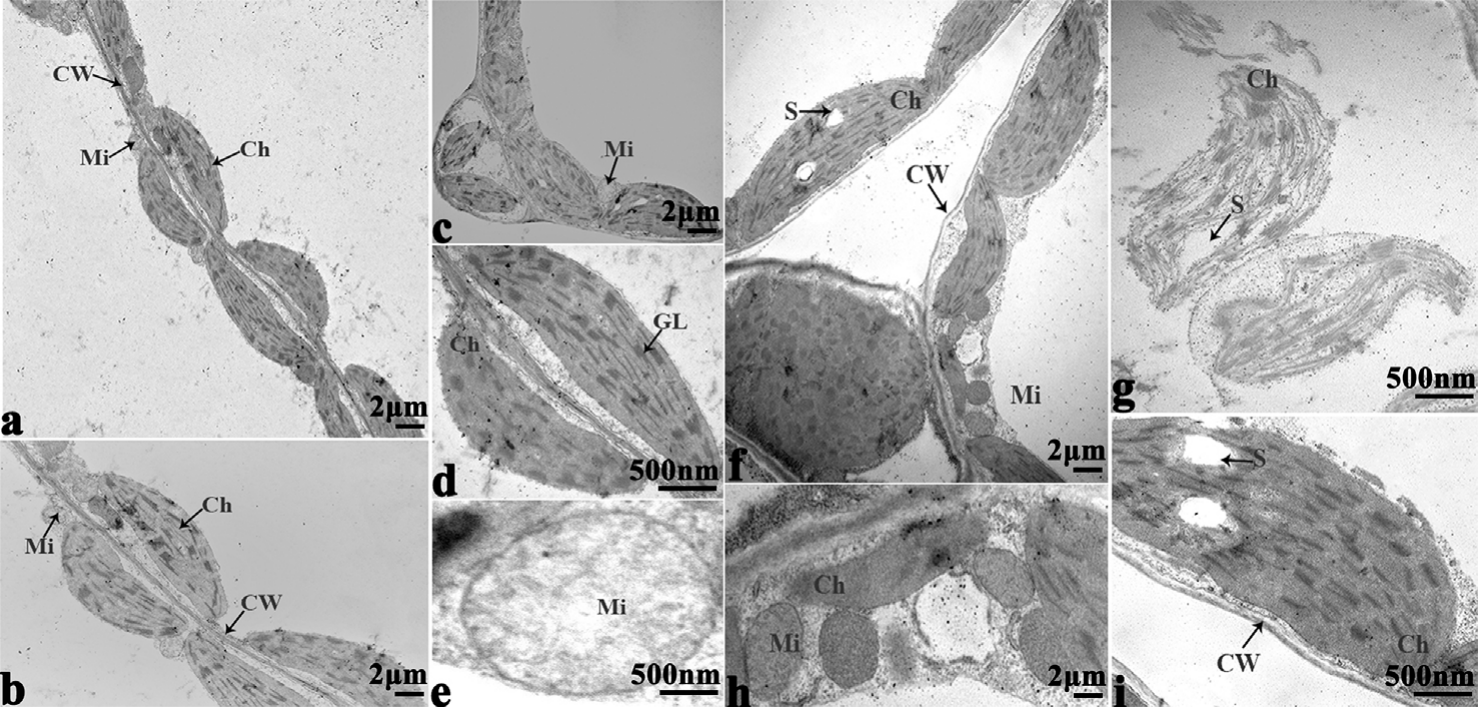
Transmission electron micrographs of leaf ultrastructure from cultivar Tianyou2238 under non-cold **(A–E)** and cold stress **(F–I)** conditions for 48 h. Mi, mitochondria; Ch, chloroplast; GL, granum lamellae; S, starch granules; OG, osmiophilic globule; CW, cytoderm. Scale bars = 2 µm and 500 nm.

In order to study the damage to the leaf mesophyll cells caused by cold stress, we investigated the leaves of 16NTS309 and Tianyou 2238 with a transmission electron microscope. Under non-cold conditions, the leaf mesophyll cells had obvious organelles that showed no significant differences between the two cultivars and the shape of each cell was regular (**Figures 9A–D** and **10A–E**). The outer envelope was clear and the chromatin inside was evenly distributed. The chloroplast shapes were lens-like and oblong, with the typical arrangement of grana and stroma thylakoids. After cold stress, more obvious alterations were observed in Tianyou2238 (**Figures 10F–I**) as compared to 16NTS309 (**Figures 9E–H**), the membrane system was not clear or complete and showed disintegration, chloroplasts were destroyed, mitochondria were swollen.

### O_2_^−^ Verification

**Figure 11:**
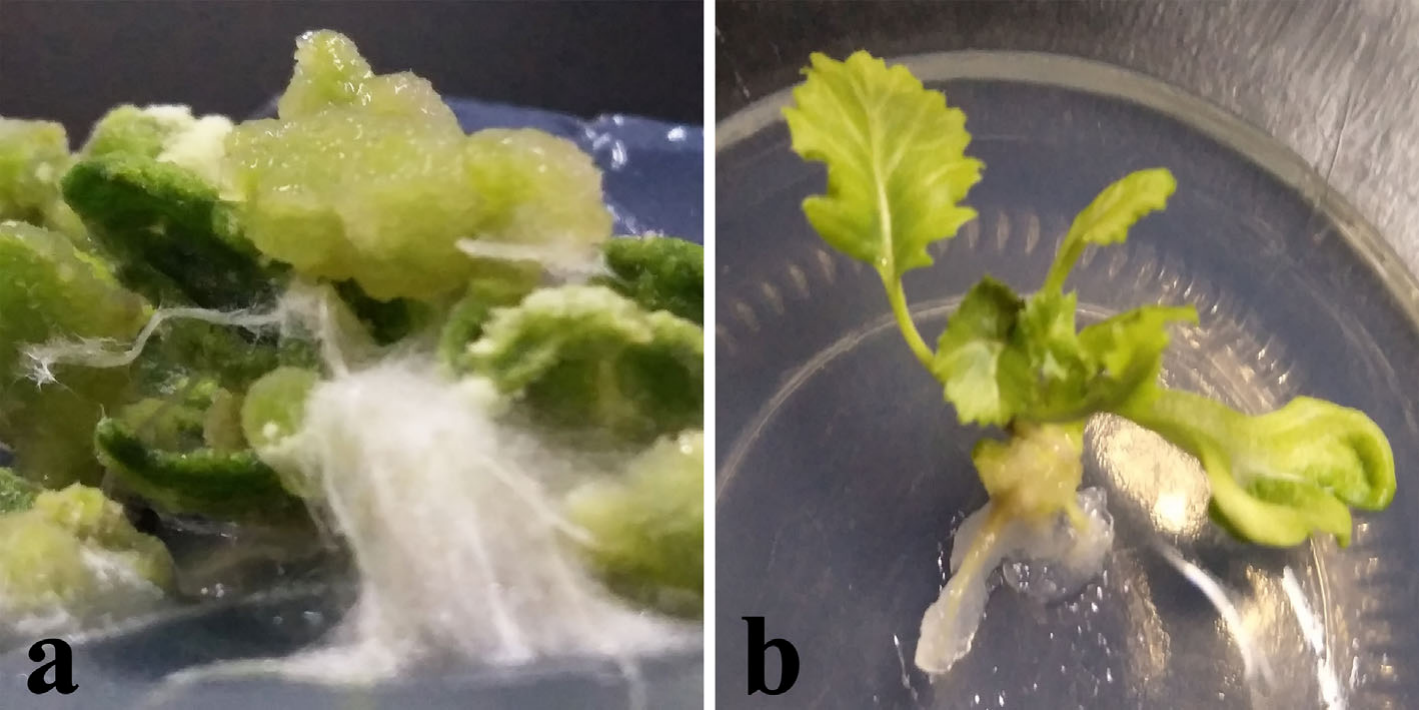
Plant callus system for leaf segments of *B. napus* cultivar Tianyou2238 (cold sensitive). To characterize callus growth in response to cold stress, calluses were grown in 200-ml flasks containing 50 ml of liquid MS medium. After four weeks incubation, the formed calluses **(a)** and plants **(b)** were divided into groups: one was maintained at 25°C as the non-cold control and the other was subjected to cold stress at 4°C.

**Figure 12:**
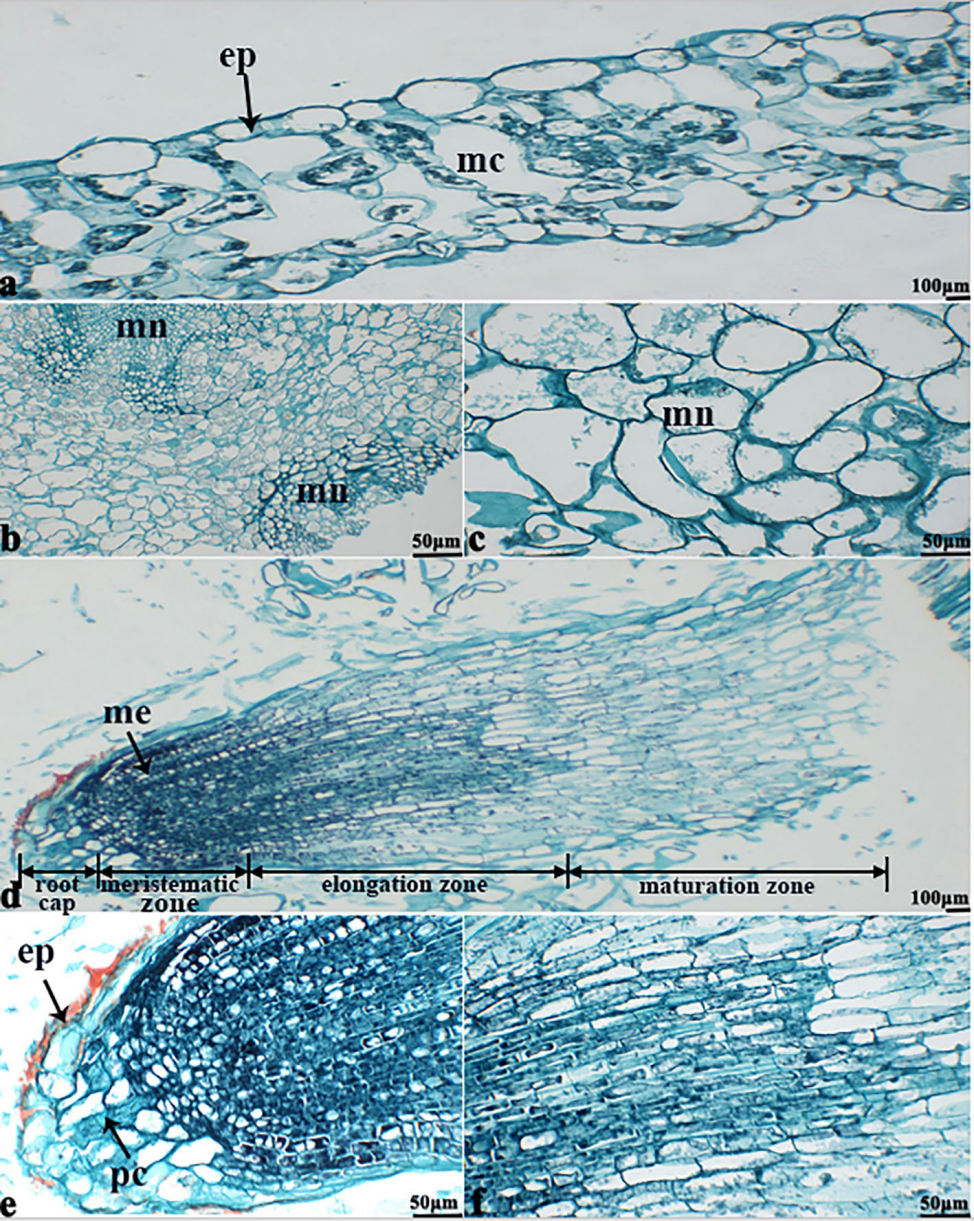
The deepest leaf **(A)**, callus **(B, C)**, root **(D–F)** samples with blue formazan precipitate areas from the callus of cultivar Tianyou2238 were sectioned for morphoanatomical analyses. ep, epidermis; mc, mesophyll cell; mn, meristematic nodule; me, meristem; pc, parenchymal cell. Scale bars = 50 µm and 100 µm.

The *in vitro* culture of plant cells and tissues allows the cytology and physiology of plants to be study (Wani et al., 2010). We established a sterile rapid propagation system for leaf segments of Tianyou2238 and used it to deeply understand the rules of O_2_^−^ production and to verify the correctness of a previous analysis (**Figure 11**). After cold stress, we found that the meristematic tissue of roots, in which the cells have mitochondria but no chloroplasts, gathered more dark blue formazan precipitate than the meristematic tissue of roots under the non-cold conditions. This result indicated that mitochondria play an active role in the production of O_2_^−^ (**Figures 12D–F**). In other mesophyll tissue cells, O_2_^−^ signals were detected mainly in the meristematic zone and cell wall (**Figures 12A–C**), which is consistent with previous results.

## Discussion

### Winter Surviving Rate and Morphology Characteristics of *B. napus*

Winter rapeseed (*B. napus*) is an important oil seed crop. As a cover crop, it helps to eliminate a dust source for the damaging sand storms in northern China. Growing winter rapeseed in dry and cold regions in northwestern China benefits economically, environmentally and ecologically (Sun et al., 2007; Sun, 2013). However, the low temperatures in the area of 35°N makes it difficult for *B. napus* varieties to survive (Sun et al., 2007; Huang et al., 2018). Our research group studied the feasibility of expanding winter rapeseed into cold regions in northwestern China and bred new lines of *B. napus* having a strong cold tolerance, which could over winter in the 36°73′N area at an altitude of 1, 517m (Pu et al., 2019b). In this study, winter surviving rate of *B. napus* variety (16NTS309) in the northwestern Lanzhou (36°73′N, altitude 1517 m) reached 85.38%, which was higher than Tianyou2238. This results indicated that 16NTS309 was a strong cold resistance, but Tianyou2238 was weaken for cold resistance. In order to adapt to low temperature, the morphology characteristics of *B. napus* are semi-erect or prostrate growth, leaves dark green or purplish red (Sun et al., 2007; Liu et al., 2013; Wang et al., 2015). The seedlings of *B. napus* of 16NTS309 tended to prostrate grow and the colors of leaves and young stems were yellow-green or purple in cold stress, which was consistent with morphological characteristics of cold tolerant *B. napus* (Sun et al., 2007; Liu et al., 2013; Wang et al., 2015; Pu et al., 2019b). Those results showed that 16NTS309 is good germplasm resources which can be used for strong cold tolerant *B. napus* breeding in northern China. To improve the cold tolerance, it is necessary to investigate the cold tolerance mechanism of winter *B. napus*.

### Physiochemical Changes of Winter *B. napus* After Cold Stress

Cold stress causes dysfunctions at the cellular level (Bowers, 1994; Shao et al., 2008). Cold tolerance in plants is correlated with multiple mechanisms, such as changes in gene expression (Örvar et al., 2000), antioxidants (Kang and Saltveit, 2001; Shao et al., 2008), accumulation of osmotic adjustment substance, which are thought to play roles in prevention of cell damage (Ristic and Ashworth, 1993). Soluble sugars (Ma et al., 2009) and proline (Lalk and Dörffling, 1985; Wanner and Junttila, 1999; Kishor et al., 2005; Shao et al., 2008) occur in the cytosol where they contribute substantially to protect plant cells from damage caused by cold stress (Gajewska and Skłodowska, 2007). In this study, we found that proline (**Figure 3D**), protein (**Figure 3E**), and soluble sugars (**Figure 3F**) markedly accumulated in response to cold stress in the *B. napus* cultivars 16NTS309 and Tianyou2238. Antioxidant systems also protect plants from ROS that are generated as a result of cold stress. SOD is an antioxidant enzyme that plays a major role in the plant defence system against oxidative stress, and it is ubiquitous in every cell of all plant types (Ali et al., 2018b). SOD catalyses the conversion of toxic O_2_^−^ radicals to H_2_O_2_ and oxygen (O_2_) (Gokul et al., 2016), and H_2_O_2_ is subsequently detoxified to water (H_2_O) by POD and CAT. We found that the cold-resistant cultivar 16NTS309 had higher SOD (**Figure 3C**), POD (**Figure 3B**), and CAT (**Figure 3A**) activity than the cold-sensitive cultivar Tianyou2238. But, when the defense system was out of adjust, excessive accumulation of ROS from multiple sources resulted in ROS bursts which caused oxidative damage to nucleic acids, lipids and proteins etc. (John et al., 2010; Barna et al., 2012; Nath et al., 2016), and damaged other surrounding cells by cold stress (Yao et al., 2001; Chen and Dickman, 2004; Min et al., 2012; Zhu et al., 2019). Studies have also shown that the permeability of cell membranes and electrolyte leakage increase resulted in an increase in the relative conductivity of tissues under low temperature (Huang et al., 2018). MDA and relative electrolyte leakage can reflect the degree of membrane damage in cold stress (Lyons, 1973; Campos et al., 2003). We found that the MDA content of 16NTS309 and Tianyou2238 under cold stress for 48 h substantially increased by 40.54% and 84.81% (**Figure 3G**), respectively. Relative electrolyte leakage (**Figure 3H**) showed rapid growth in Tianyou2238. This result showed that ROS induced lipid peroxidation and the level of injury depended on the cold-resistant properties of the cultivars. Our findings are in agreement with previous study that cold-tolerant species, such as *Santalum album* (Zhang et al., 2017), *Vitis amurensis* (Xu et al., 2014), *Solanum lycopersicum* (Chen et al., 2015), *B. rape* (Ma et al., 2019), and *B. napus* (Pu et al., 2019a) have strong protective enzyme activity and high content of soluble regulators which can reduce ROS production. The present physio-chemical study confirmed that cultivar 16NTS309 had significantly higher cold resistance than cultivar Tianyou2238.

### The Variation of Cell Ultrastructure Alterations Caused by Cold Stress

The present studies showed that cold stress of winter *B. napus* is associated with pronounced modifications in the ultrastructure of leaf cells. Wise et al. (1983) reported that ultrastructural changes of cold-sensitive species are thought to be more pronounced and induced more quickly in plants. In the present study, more obvious ultrastructural alterations in leaf mesophyll including the damage of membrane system, destruction of chloroplast and swelling of mitochondria were observed in cold-sensitive variety of Tianyou2238 compared to cold-resistant cultivar 16NTS309. Similar damage in the leaf mesophyll cell ultrastructures was investigated in *B. napus* against cold stress (Stefanowska et al., 2002; Pu et al., 2019a). Previous research reported that chloroplasts are thought to be the first and most severely affected organelle (Kratsch and Wise, 2000), and cold stress may lead to unstacking of grana and disintegration of the chloroplast envelope (Klein, 1960; Kislyuk, 1963; Jagels, 1970; Taylor and Craig, 1971; Murphy and Wilson, 1981; Kratsch and Wise, 2000; Vella et al., 2012). In our study, cold stress affected more obviously in chloroplast shape and caused more significant ultra-structure changes in weak cold resistant variety Tianyou2238 than cold-resistant cultivar 16NTS309. The changes of chloroplast shape also occurred in chilling-sensitive plants such as mung bean (Ishikawa, 1996) and *Arabidopsis* (Ristic and Ashworth, 1993). Mitochondrial shape and internal composition changes including mitochondrial swelling and loss of cristae within mitochondria also occurred in *B. napus* responding to plant chilling in cold-sensitive plants (Ilker et al., 1976; Murphy and Wilson, 1981; Ishikawa, 1996; Lee et al., 2002) (Stefanowska et al., 2002; Pu et al., 2019a). The alterations of mitochondrial ultrastructure were observed more in Tianyou2238 while less in cold-resistant variety of 16NTS309 after cold stress, which were consistent with those reported previously.

### Sources of O_2_^−^

**Figure 13:**
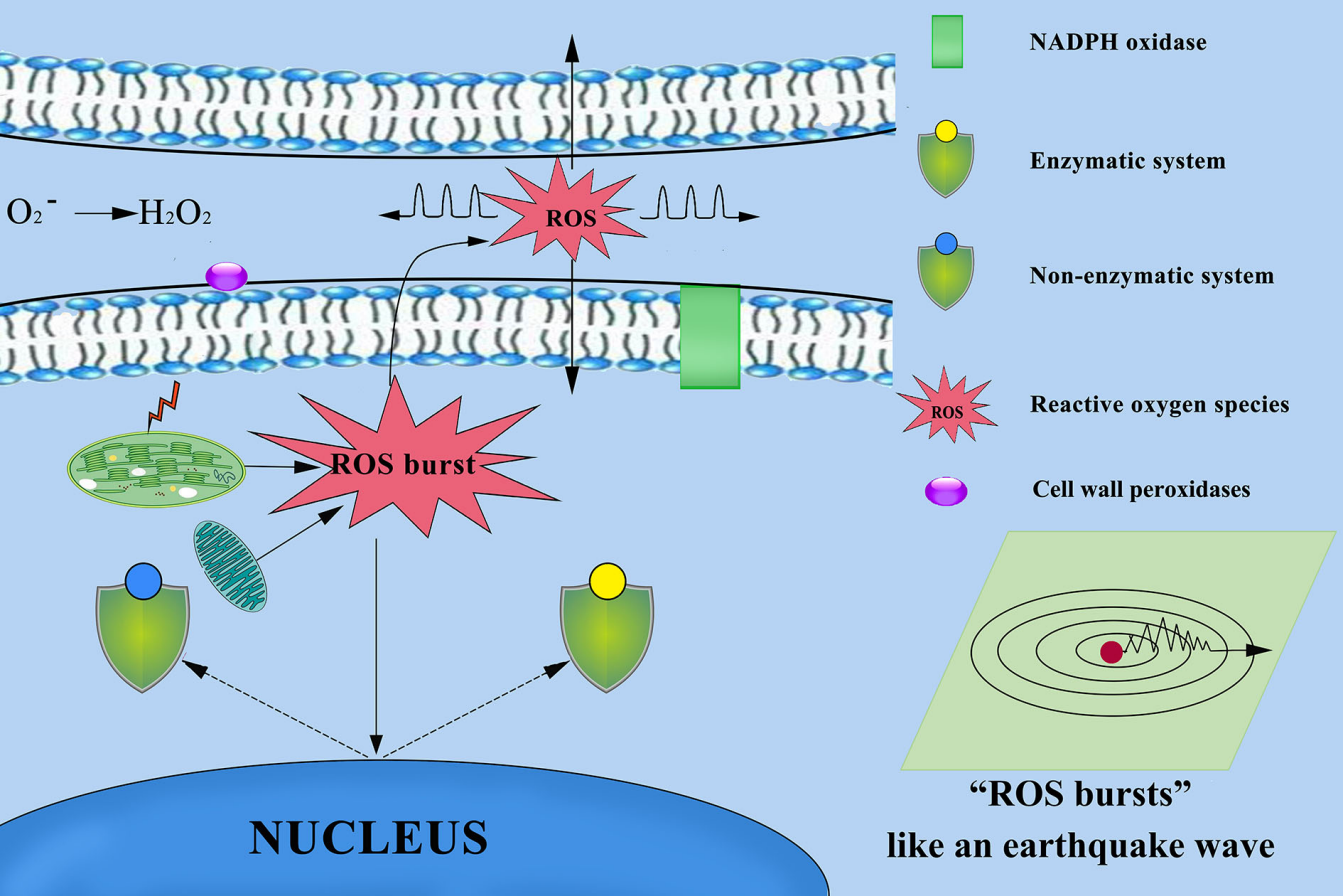
Mechanism of reactive oxygen species (ROS) diffusion in cytoplasmic and other cells. Chloroplasts, mitochondria, cell wall peroxidases, and plasma membrane NADPH oxidases play important roles in ROS production. ROS as highly dynamic signalling molecules that ROS propagate throughout the different tissues and cells, and between different organelles and cells over long distances. When the plant defence system collapses, excessive accumulation of ROS from multiple sources results in ROS bursts that damage other surrounding cells and severely damage cellular structures.

ROS signals are known to form signatures during the biotic and abiotic stress responses of plants. For example, there is a quick release of ROS, called an oxidative burst, following stress, which is an important signature not only for the local immune response but also for cell-to-cell communication (Miller et al., 2009; Miller et al., 2010). In both cultivars, the O_2_^−^ signals were distributed mainly in cambium tissue, meristematic cells, mesophyll cytoplasm, and surrounding the cell walls of root (**Figures 5C, D, G, H**), stem (**Figures 6C, D, G, H**), leaves (**Figures 7B, D**), and leaf vein (**Figures 8B, D**), but the quantities varied. This phenomenon can be explained visually as follows (**Figure 13**). O_2_^−^ are produced in the cell wall and are correlated with cell wall peroxidases (Bindschedler et al., 2006) and plasma membrane NADPH oxidases (Torres et al., 2002; Suzuki et al., 2011). These enzymes can translocate electrons across the plasma membrane and reduce extracellular oxygen to yield O_2_^−^ in the cell wall. The intracellular production of a lot of O_2_^−^ (Zurbriggen et al., 2010; Singh et al., 2019; Qi et al., 2020) may be because organelles with high oxidizing metabolic activity or with an intense rate of electron flow, such as chloroplasts and mitochondria (Mittler et al., 2004; Yao and Greenberg, 2006), work to produce O_2_^−^ in the mesophyll cytoplasm (Liu et al., 2007; Zurbriggen et al., 2009; Zurbriggen et al., 2010) of plant cells. Our studies found that the meristem of the root, which has mitochondria but no chloroplasts, produced more O_2_^−^ than the other tissues. The mitochondria respiratory chain continuously produces O_2_^−^ because of the numerous electron transfer reactions that take place in the presence of oxygen (**Figures 12D–F**). These results make it clear that mitochondria produce O_2_^−^. A greater O_2_^−^ content accumulated in palisade tissue and mesophyll cells compared with that in other leaf cells. This result indicated that chloroplasts play an active role in the production of O_2_^−^. These findings confirm previous reports that chloroplasts and mitochondria play important roles in O_2_^−^ production.

### O_2_^−^ Was a Highly Dynamic Signalling Molecules

ROS exists in different forms with significantly different molecular properties (Schmidt and Schippers, 2015). For example, short-lived O_2_^−^ are unable to passively through the membrane’s lipid bilayer (Mittler et al., 2011). In addition, O_2_^−^ could be easily converted into hydrogen peroxide (H_2_O_2_) by variety of reactions (Gilroy et al., 2016; Toyota et al., 2018; Singh et al., 2019) that readily transfers across membranes passively. Previous study reported that O_2_^−^ depending on the cellular environment can travel 50–500 nm (Saran and Bors, 1989; Pitzschke et al., 2006; Schmidt and Schippers, 2015). Our studies also found that the root structure of cambium, which appeared O_2_^−^ bursts, O_2_^−^ signals can spread throughout cells in different directions (**Figure 5H**). We established a sterile rapid propagation system for leaf segments of Tianyou2238 and proved O_2_^−^ bursts were appeared in the cells of meristematic zone and O_2_^−^ gradually increased in the cells of elongation zone (**Figure 12D**). Thus, those results strengthen the view of O_2_^−^ as highly dynamic signaling molecules. They also found the existence of the auto-propagating characteristics of the ROS wave and ROS can long distance auto-propagating signals (Takeda et al., 2008; Mittler et al., 2011; Singh et al., 2019). Based on this study speculated that not only ROS wave presented in *B. napus*, but also ROS signaling as a dynamic process that occurs within cells between different organelles, as well as between cells over long distances (**Figure 13**). ROS signals are known to form signatures during the biotic and abiotic stress responses of plants. During normal metabolic processes, plant cells produce a variety of ROS, including the O_2_^−^, H_2_O_2_ and hydroxyl radicals (O’brien et al., 2012) and there is a quick release of ROS, following cold stress, which is an important signature not only for the local immune response but also for cell-to-cell communication (Miller et al., 2009; Miller et al., 2010; Ma et al., 2019; Qi et al.,2020).

## Conclusion

In our study, physiological, biochemical, and cytological methods were used to investigate the plant’s response to cold stress in two *B. napus* cultivars 16NTS309 and Tianyou2238. The results showed that cold-tolerant *B. napus* of 16NTS309 has strong protective enzyme activity and high content of soluble regulators, which can increase the cold resistance and reduce the production of ROS. More obvious ultrastructural alterations in leaf mesophyll were observed in Tianyou2238 compared to 16NTS309. From the above discussion and findings, it is concluded that 16NTS309 cultivar is cold-tolerant and Tianyou2238 cultivar is cold-sensitive. We also found that in both cultivars O_2_^−^ signals were distributed mainly in cambium tissue, meristematic cells, mesophyll cytoplasm, and surrounding the cell walls of root, stem, leaves, and leaf vein by morphoanatomical analysis, while the quantities varied. These results strengthened the view of O_2_^−^ as highly dynamic signaling molecules. This study shed light on the physiological, biochemical cytology changes associated with the response to cold stress of *B. napus* which would be helpful in cold-resistant varieties breeding. Further studies will be necessary to clarify the regulating mechanisms of 16NTS309 in cold response.

## Data Availability Statement

All datasets generated for this study are included in the article/supplementary material.

## Author Contributions 

WQ and WS conceived and designed the study. WQ, WF, LM, SL, and PW conducted the experiments. WQ, WS, ZQ, SL, CC, and CY analyzed the data. JW, ZQ, and YW contributed reagents, materials, and analysis tools. WQ wrote the manuscript. We thank Margaret Biswas, PhD, from Liwen Bianji, Edanz Group China (www.liwenbianji.cn/ac), for editing the English text of a draft of this manuscript. “Sections of this manuscript are released as a pre-print at [Reactive oxygen species as important regulators of cell division. *bioRxiv.* doi: https://doi.org/10.1101/2020. 03. 06. 980474], (Qi, W., Ma, L., Wang, F., Wang, P., Wu, J., Wang, J., et al.)”.

## Conflict of Interest

The authors declare that the research was conducted in the absence of any commercial or financial relationships that could be construed as a potential conflict of interest.
